# Campylobacter bacteremia in a Child With Nephrotic Syndrome: A Case Report

**DOI:** 10.7759/cureus.91320

**Published:** 2025-08-31

**Authors:** Matthew S Kreitman, Maya Heled Akiva, Evgenia Gurevich

**Affiliations:** 1 Pediatric Department, Barzilai University Medical Center, Ashkelon, ISR; 2 Pediatric Infectious Disease Department, The Edith Wolfson University Medical Center, Holon, ISR; 3 School of Medicine, Tel-Aviv University, Tel-Aviv, ISR; 4 School of Medicine, Ben-Gurion University of the Negev, Be'er-Sheva, ISR

**Keywords:** antibiotic selection, campylobacter bacteremia, case report, immunosuppression, nephrotic syndrome

## Abstract

*Campylobacter* spp. bacteremia can be seen in immunocompromised patients. Nephrotic syndrome (NS) predisposes patients to serious bacterial infections (SBI). The most common SBIs in NS are spontaneous bacterial peritonitis (SBP), sepsis, and bacteremia due to *Streptococcus* *pneumoniae* or *Escherichia* *coli*. To date, despite several case reports in adults, there has been no report of *Campylobacter bacteremia* in the context of pediatric NS. A seven-year-old boy with NS receiving steroid treatment was admitted to our hospital, presenting with fever, diarrhea, and abdominal pain. Ceftriaxone was initially prescribed as empirical therapy for the usual suspected bacteria causing SBP. Unexpectedly, blood culture growth of *Campylobacter jejuni *was identified, and antibiotic therapy was altered to azithromycin accordingly. The patient responded well to treatment, repeated cultures were negative, and he was discharged once afebrile and well. The clinical significance of this case underscores the importance of a high index of suspicion for an atypical bacterial pathogen, which may need alternative antibiotic therapy.

## Introduction

*Campylobacter* spp. is well known as a bacterial pathogen causing acute gastroenteritis, but, in certain cases, it may cause bacteremia. The latter complication is thought to be due to multiple factors, including bacterial translocation, invasion, and evasion of the immune system. Thus, *Campylobacter bacteremia *is commonly seen in patients with immunodeficiency, immunosuppression, and intestinal mucosal compromise secondary to malnutrition. In a retrospective, population-based study conducted between 1989 and 2010 in Southern Israel, 76 campylobacteremia episodes (one per child per month) were identified, both in previously healthy and more commonly in immunocompromised children [1].

Idiopathic nephrotic syndrome (INS) is characterized by massive proteinuria, hypoalbuminemia, and edema and predisposes the patient to bacterial infection due to urinary loss of immunoglobulins and complement system proteins, as well as corticosteroid (CS) treatment. INS patients usually respond to CS, which indicates a good prognosis, but tend to relapse in many cases. The clinical course with relapse occurring within two weeks of discontinuation of CS treatment or during tapering down is defined as steroid-dependent NS and usually requires the use of steroid-sparing immunosuppressive medications to prevent adverse effects of CS [2].

The most serious bacterial infection (SBI) among children with INS is spontaneous bacterial peritonitis (SBP). Peritonitis may be accompanied by sepsis or bacteremia, most commonly by *Streptococcus pneumoniae *and Gram-negative *Enterobacteriaceae *spp. such as *Escherichia coli *[3]. In a retrospective study, the frequency of SBI in children with NS admitted to a tertiary pediatric hospital in Israel was estimated as 14.7% [4]. To date, there has been no report of *C. bacteremia* in the context of pediatric NS. Herein, we describe the diagnosis and treatment of *C. bacteremia *in a child with NS.

## Case presentation

A seven-year-old boy with steroid-dependent NS (SDNS), treated for five days with CS (oral prednisone 30 mg q12h) for NS relapse, presented to our medical center with fever and abdominal pain two days after diarrhea. He had no recent contacts with ill persons or animals and did not recently travel. He was diagnosed with NS six months before this admission and had already had three relapses, appearing shortly after CS treatment discontinuation and fully responding to CS within two weeks. On admission, his temperature was 39 degrees Celsius, with normal blood pressure, heart rate, and oxygen saturation. His BMI (based on his pre-relapse weight) was 18.8 (BMI percentile: 88), indicating obesity as a treatment side effect. Physical examination was remarkable for generalized edema, but the abdomen was soft with no tenderness or peritoneal signs. Laboratory investigations (Table 1) reflected his NS relapse. Based on the clinical presentation of fever, abdominal pain, and elevated inflammatory markers in a nephrotic patient, SBP was suspected, and, accordingly, blood cultures were drawn, and the patient was hospitalized. Empirical therapy with intravenous ceftriaxone was initiated in parallel with corticosteroids (prednisone 30 mg q12h) for NS relapse. After 24 hours, he was afebrile, and abdominal symptoms had resolved. Unexpectedly, blood culture growth was reported with a Gram stain revealing Gram-negative rod bacteria. Blood culture was repeated 24 hours after the initial sample, and parenteral ceftriaxone was continued. The following day, identification from the first blood culture revealed *Campylobacter jejuni* as the pathogen, sensitive to macrolides and tetracycline and resistant to ciprofloxacin. Antibiotic therapy was then switched to intravenous azithromycin, along with prednisolone, esomeprazole, and vitamin D. The patient remained afebrile and asymptomatic throughout the treatment. Once two consecutive daily drawn cultures were confirmed negative, he was discharged with oral azithromycin for a total course of seven days, and corticosteroid therapy was concomitantly continued until he entered full NS remission after an additional two weeks, along with vitamin D and calcium to prevent the osteoporotic side effects of steroids.

**Table 1:** Laboratory findings of SDNS patient upon admission SDNS: steroid-dependent nephrotic syndrome, WBC: white blood cells, CRP: C-reactive protein, IgG: immunoglobulin G, U P/Cr: urine protein-creatinine ratio

Variables	Results	Normal value
WBC (1,000/µL)	13.8	5-15
n Neutrophils (%)	11.7 (84.4)	
n Lymphocytes (%)	1.3 (9.6)	
n Monocytes (%)	0.8 (5.9)	
CRP (mg/dL)	1.7	<0.5
Albumin (g/dL)	2.2	3.5-5
IgG (mg/dL)	391	443-1095
U P/Cr (mg/mg)	7.3	<0.2

## Discussion

*C. bacteremia* is rare and may rarely manifest as a complication of gastroenteritis. In the immune-compromised patients, it is significantly more common and presents more often in older children and commonly without gastrointestinal symptoms or as recurrent episodes [1]. The pathophysiologic mechanisms include bacterial translocation, invasion, and evasion of the immune system [1]. In patients with nephrotic syndrome, several mechanisms contribute to the immunocompromised state, particularly immunosuppressive treatment, urinary loss of complement system proteins, and hypogammaglobulinemia [5]. Such immunocompromise, among other factors, may render children with NS at increased risk of *C. bacteremia* [6]. Our patient with NS relapse had documented low immunoglobulin G levels and received CS treatment, leading to immunocompromise. The most likely mechanism of *C. bacteremia* in our patient was bacterial translocation from the intestinal lumen at the time of gastroenteritis (our patient had diarrhea two days prior to his admission).

Appropriate antimicrobial therapy in *C. bacteremia *is associated with improved 30-day survival [7]. Considering that, in a past study, only 1.6% of isolates were sensitive to ceftriaxone, and over a quarter of the *Campylobacter *blood isolates were resistant to ciprofloxacin [8], according to the study of campylobacteremia in pediatric patients conducted in our region where all isolates tested for antibiotic susceptibility were sensitive to macrolides, macrolides are the preferred antibiotic choice [1].

In our patient, ceftriaxone, a reasonable empirical choice of antibiotic in such cases presenting with fever and abdominal pain, did not give proper coverage for the isolated *Campylobacter*, which was susceptible to macrolides. Thus, in cases of fever, following acute gastroenteritis in a nephrotic patient, *Campylobacter* infection should be suspected, and appropriate antibiotic therapy should be given.

## Conclusions

This is the first report of *C. bacteremia *in a pediatric patient with INS. The clinical significance of this case underscores the importance of a high index of suspicion for an atypical bacterial pathogen, which may require alternative antibiotic therapy.
